# Efficacy of an Integrative Treatment for Tinnitus Combining Music and Cognitive-Behavioral Therapy—Assessed With Behavioral and EEG Data

**DOI:** 10.3389/fnint.2020.00012

**Published:** 2020-04-07

**Authors:** Tianci Feng, Mingxia Wang, Hao Xiong, Yiqing Zheng, Haidi Yang

**Affiliations:** ^1^Department of Otolaryngology, Sun Yat-sen Memorial Hospital, Sun Yat-sen University, Guangzhou, China; ^2^Hearing and Speech Science Department, Xinhua College of Sun Yat-sen University, Guangzhou, China

**Keywords:** alpha wave, phantom percept, CBT, lateral inhibition, sLoreta

## Abstract

Chronic tinnitus is a prevalent condition that could cause severe negative impact on an individual’s life. However, there has not been an established treatment due to a limited understanding of the pathophysiology of this multifarious disorder. In this study, we tested the efficacy of an integrative treatment, combining music therapy with cognitive-behavioral therapy (CBT). We collected three groups of patients receiving three different treatments: Music-CBT, music therapy and CBT. We used both subjective (i.e., questionnaires) and objective (i.e., resting-state EEG data) measurements to assess the behavioral and neural changes brought upon by the treatments. Analyses of the subjective measurements found a significant improvement of scale scores in Music-CBT and CBT, but not in the Music group. Analysis of the EEG data further showed increased powers in alpha and theta band after the Music-CBT treatment, and increased gamma power after CBT, whereas no significant difference was found for the music therapy. Further source localization analysis of alpha and theta changes in the Music-CBT group found that primary sources of the changes were located at auditory processing regions such as superior temporal gyrus, and higher emotional and cognitive processing regions such as ventromedial prefrontal cortex (vMPFC), lateral prefrontal cortex and parahippocampus. These results indicated that Music-CBT was effective in improving tinnitus symptoms on both a behavioral and neural level, which is more robust than the music therapy or CBT alone.

## Introduction

Subjective tinnitus (hereinafter referred to as tinnitus) is the perception of sound without corresponding external stimulus (Jastreboff, 1990). It is one of the most prevalent symptoms of hearing disorders (Lockwood et al., 2002; Heller, 2003; Langguth, 2015), affecting over 10–15 percent of the adult population (Jastreboff, 1990), among whom an estimated 5%–15% of the condition can become chronic and have a significant negative impact on the patients’ emotional state and quality of life (Dobie, 2003; Heller, 2003; Bhatt et al., 2016). Although various treatments have been developed, efficacies of many of them were limited, and no established treatment has been found, which is largely due to a limited understanding in the pathophysiology of this condition.

In recent years, increasing numbers of researchers have agreed upon a theory that tinnitus originated from cochlear damage, which progressed into a chronic condition due to compensatory enhancement of central auditory activities (Auerbach et al., 2014). Specifically, a reduced cortical inhibition in the auditory cortex, especially the deafferented areas corresponding to the damaged cochlear regions, resulted in hyperactivity of the auditory cortex and an over-representation of neighboring frequencies (Weisz et al., 2006; Rauschecker et al., 2010). However, it has been observed that moderate exposure to certain sound could induce a temporary suppression of tinnitus, a phenomenon called “residual inhibition” (Feldmann, 1971; Roberts, 2007). This could be related to the finding that moderate sound exposure could slow the degeneration of cochlear hair cells and nerve fibers and reduce cortical reorganization after hearing loss (Willott and Turner, 1999; Willott and Bross, 2004; Noreña and Eggermont, 2006).

Tinnitus masking is a therapy developed based on the above findings (Pienkowski, 2019). This therapy primarily involves using various sound stimulations, such as broadband/narrowband noise to completely or partially mask the tinnitus (tinnitus masking). It has shown promising results in some studies (Formby and Keaser, 2007; Henry et al., 2015). For instance, Henry and colleagues tested the efficacy of hearing aids with built-in noise/sound generators for tinnitus patients, and found that compared with the control group whose noise feature of the instruments were deactivated, the experimental group with their noise generator activated had a seemingly larger improvement in tinnitus symptoms (Henry et al., 2015). However, there are also controversies regarding whether improvements could be caused not by the sound stimulations *per se*, but the psychological counseling that is usually incorporated in the therapy (McKenna and Irwin, 2008). For instance, a study compared the outcomes of three types of tinnitus treatment: tinnitus retraining therapy (which is a combination of tinnitus masking and counseling), tinnitus masking and counseling, and found that improvement was seen in questionnaire scores for all three treatment groups, and no difference was found among these groups (Formby and Keaser, 2007).

The explanation for the limited efficacy of tinnitus masking could be that this primarily bottom-up approach alone cannot fully address a complex situation such as tinnitus. Tinnitus is a subjective symptom, which is found to be related to not only the acoustic characteristics of the phantom sound (House and Brackmann, 1981; Andersson, 2003; Cima et al., 2011), but also top-down modulations such as cognitive misinterpretations, negative emotional reactivity, and dysfunctional attentional processes (Erlandsson and Hallberg, 2000; Andersson and McKenna, 2006; Langguth et al., 2011). The sustaining of tinnitus could involve a brain mechanism much more sophisticated than merely maladaptive hyperactivities in the auditory cortex (Colder and Tanenbaum, 1999; Leaver et al., 2011; De Ridder et al., 2014; Elgoyhen et al., 2015). In other words, the production and sustaining of bothersome tinnitus is likely to be a result of both bottom-up and top-down processes.

A better approach to address these two processes underlying tinnitus could be an integrative treatment, combining a bottom-up approach, such as tinnitus masking, with a top-down approach, such as cognitive-behavioral therapy (CBT; Cima et al., 2014). CBT is another popular treatment which utilizes a series of operations including education, psychological counseling and mindfulness practice to alleviate the negative psychological impact that has reinforced the persistence of the symptoms (Cima et al., 2014). It has been shown to be able to reduce distress associated with tinnitus, and improve quality of life and daily functioning (Andersson and Lyttkens, 1999; Andersson, 2002; Sadlier et al., 2008; Martinez-Devesa et al., 2010; Cima et al., 2014). It is our hypothesis that by combining the two approaches, it is possible to simultaneously retrain the damaged auditory system and readjust the cognitive, emotional and attentional functions subsequently altered by the damage, to fully optimize the rehabilitation process.

The objective of this study is to test the efficacy of a novel treatment combining tinnitus masking with CBT. Music was chosen as the stimulation sound for tinnitus masking, because music therapy has shown beneficial effects in various medical applications (Nickel et al., 2005) and is currently clinically used in tinnitus treatment (Kochkin et al., 2011). We hypothesized that the Music-CBT should show the most robust effect in improving tinnitus symptoms, with respect to treating patients with music masking or CBT alone. To test our hypothesis, three groups of tinnitus patients were treated with three different approaches: Music-CBT, music therapy and CBT. Before- and after-treatment measurements were collected, with Tinnitus handicap inventory (THI) and subjective anxiety scale (SAS) as behavioral assessments, and resting-state EEG recordings as neural assessments. Source localization analyses of the EEG data were subsequently performed for further investigation into spatial patterns of the neural change brought upon by the treatment.

## Materials and Methods

### Participants

A total of 56 tinnitus patients were recruited from the Otolaryngology Clinic, Sun Yat-sen Memorial Hospital, Sun Yat-sen University. Detailed selection criteria for inclusion and exclusion in this study are as follows:

(1) Subjective tinnitus as a sole complaint;
(2) Tinnitus frequency between 125 and 8,000 Hz;
(3) Tinnitus duration over 3 months;
(4) No prior tinnitus treatment taken;
(5) No ototoxic drugs taken, such as gentamicin;

Forty-four of the 56 patients were collected prospectively for the Music-CBT and music therapy, while 12 patients treated with CBT were retrieved retrospectively from previous data. For the Music-CBT and music therapy, the 44 patients were assigned alternately into one of the two treatment groups, resulting in 22 patients in each group.

All patients underwent the same procedures for an otoscopy examination and pure-tone audiometry. For tinnitus matching, pure tones of 125–8,000 Hz, as well as narrowband noise and white noise were used to compare the tonality and loudness balance of the tinnitus.

A written consent form was obtained from every participant before the experiment. The study was approved by the Institution Review Board of the Sun Yat-sen Memorial Hospital at Sun Yat-sen University of China.

### Procedure and Stimuli

Each patient underwent a behavioral test and an EEG test right after he/she was enrolled for the study (before-treatment tests, BT), and received the same two tests 3 months after the treatment (after-treatment tests, AT).

#### The Music Therapy Procedure

The procedure of tinnitus masking with music was the same for both the Music therapy and Music-CBT groups. One treatment session was performed by a trained clinician right after the patient was enrolled in the study. In this session, the patient sat in a soundproof booth while listening to some light music chosen by the patient, from a list of music built in a system called TinniTestTM (Micro-DSP Technology Company Limited). The volume of the music was controlled within 10 dB above their tinnitus loudness, which is just enough to mask their tinnitus. This session was meant as an example for the patients to follow after they returned home.

Subsequent treatments were performed by the patients themselves at home. Patients were instructed to listen to some light music of their own choice, for more than 2 h per day. They were advised to listen to the music early in the morning or afternoon. Loudness of the music should be similar to that in the first session, which is just enough to mask their tinnitus, without causing any discomfort. However, given the variability of tinnitus loudness during the day, volume of the music was not specifically controlled. Follow-ups were done through phone-calls or online social apps at approximately once a week.

#### The CBT Procedure

The CBT procedures were the same for both the Music-CBT and CBT groups. One treatment session of CBT was performed right after the patient was enrolled in the study. During this session, the clinician first provided tinnitus-specific education and counseling to correct misunderstandings for tinnitus, lower the anxiety associated with the symptoms as much as possible, and establish trust between the clinician and the patient.

Mindfulness training is a major component of CBT, therefore during the first session, the clinician also provided for the patient demonstration on how to perform mindfulness practice at home. The patients were instructed to sit or lie in a quiet place, close their eyes, clear their minds, try to focus on their breathing and relax. Patients were required to practice mindfulness training for 10–15 min every day, preferably right before or after they go to bed. Follow-ups were done through phone-calls or online social apps at approximately once a week.

#### The Music-CBT Procedure

The Music-CBT procedures are a combination of the above two treatments. Same as the other two treatments, one session was performed right after the patients were enrolled, to set as an example for them to follow at home. During this first session, the clinician provided both demonstration of the music masking operations, as well as education and counseling and mindfulness practice, which were as follows:

(1) Education as well as psychological counseling was first provided, in order to correct misunderstandings for tinnitus, lower the anxiety associated with the symptoms as much as possible, and to establish trust between the clinician and the patient.
(2) Music therapy was applied in the same way as in the Music group, in which the patients were required to listen to some light music for at least 2 h per day in a proper volume.
(3) Each patient was required to practice mindfulness training for 10–15 min per day, preferably right before or after sleep. The patients were instructed to sit or lie in a quiet place, close their eyes, clear their minds, try to focus on their breathing and relax.

Follow-ups were done through phone-calls or online social apps at approximately once a week.

#### Behavioral Measurements

In this study, we used two scales as behavioral assessments for symptom severity: the THI and SAS. The THI is a widely used questionnaire to measure tinnitus severity, which was developed by Newman et al. (1996). It contains 25 items, and scores can be graded into five levels of symptom severity ranging from slight to catastrophic. The SAS scale is a 20-item questionnaire developed by Zung (1971), designed to quantify anxiety levels in patients based on scoring in four groups of manifestations: cognitive, autonomic, motor and central nervous system symptoms. This scale was chosen under the consideration that anxiety is a major symptom within tinnitus patients (Pattyn et al., 2016).

#### EEG Data Collection

Continuous resting-state EEG recordings were made using a 128-channel dense-array system (HydroCel Geodesic Sensor Net; Electrical Geodesics Inc., OR, USA; see Supplementary Figure S1 for the electrode positions). Sampling rate was set at 1,000 Hz and band-pass filtered at 0.1–100 Hz. The CZ electrode was used as reference for online recording. Impedances were kept below 50 KΩ for all electrodes. A resting EEG was obtained over approximately 7 min with eyes closed.

### Data Analysis

#### Demographic and Clinical Data Analysis

Data were analyzed using SPSS 22.0. One-way ANOVAs were performed to compare differences among the three treatment groups in age, disease duration, tinnitus loudness and hearing threshold. Gender and laterality of tinnitus were analyzed with non-parametric tests.

#### Behavioral Data Analysis

Two Split-Plot ANOVAs were performed for the two subjective scales, THI and SAS, respectively, with a between-subject variable of Group (Music-CBT, Music and CBT) and a within-subject variable of TimeOfTest (BT and AT). Homogeneity of variance was tested with the Levene’s test. Simple effect analyses were performed when significant interactions were found.

In the above analyses, Bonferroni procedures were used to control for multiple comparisons, in which an adjusted alpha level of 0.025 (which is 0.05 divided by two, the number of tests) was used for a *p*-value to be considered significant.

#### EEG Data Analysis

##### Preprocessing

EEG data were processed with the EEGLAB toolbox implemented in MATLAB13.0 (Delorme and Makeig, 2004). The data was further down-sampled to a rate of 500 Hz, re-referenced to bilateral mastoids, and band-pass filtered at 0.5–45 Hz. A notch filter was implemented at 50 Hz to reject the power line interference. The continuous data was visually inspected for channels or time epochs containing high-amplitude, high-frequency muscle noise, and other irregular artifacts. After manual removal of these gross artifacts, an Independent Component Analysis (ICA) was performed to remove saccades, muscle movements, eye movements, and physiological noises such as heartbeats. All 126 channels (two channels were used as reference channels) were used in the subsequent analyses of this study.

##### Spectral Power Calculation

After preprocessing, the artifact-free EEG recordings were divided into 2-s epochs. The spectral density was calculated with Fast Fourier Transform (FFT) and averaged over all the epochs. Absolute power spectra values at each electrode were calculated for the following five frequency bands: Delta (0.5–3.5 Hz), Theta (4–7.5 Hz), Alpha (8–13 Hz), Beta (14–30 Hz) and Gamma (31–44 Hz).

##### Statistical Analysis

Five Split-Plot ANOVAs were performed for power values of the five frequency bands respectively, with a between-subject variable of Group and a within-subject variable of TimeOfTest. Homogeneity of variance was tested with the Levene’s test. Simple effect analyses were performed when significant interactions were found.

In the above analyses, Bonferroni procedures were used to control for multiple comparisons, in which an adjusted alpha level of 0.01 (which is 0.05 divided by five, the number of bands) was used for a *p*-value to be considered significant.

##### Source Localization Analysis

Given that significant BT vs. AT differences were found in alpha and theta powers for the Music-CBT treatment, a source localization analysis was further performed in order to investigate the spatial characteristics of these changes, using the sLORETA-KEY software package (Pascual-Marqui, 2002). The sLoreta approach allows us to calculate the signal source in low-resolution brain images with no localization error (Greenblatt et al., 2005; Sekihara et al., 2005). In the current sLoreta software, the spatial resolution is 5 × 5 × 5 mm, which is equivalent to 6,239 voxels. 3D current source were estimated based on the Talairach Daemon (Lancaster et al., 2000). Before- and after-treatment alpha and theta values were first normalized, and then analyzed for the Music-CBT using paired sample *t*-tests and non-parametric permutations (5,000 times). Results were demonstrated using a Montreal Neurologic Institute average MRI brain (MNI152; Mazziotta et al., 2001).

## Results

### Demographic and Clinical Analysis

As seen in Table 1, no difference was found in age, gender, duration, hearing threshold or laterality of tinnitus among the three groups. However, there was a significant difference found in tinnitus loudness. *Post hoc* pairwise comparisons revealed that the CBT vs. Music contrast contributed to this difference, in which the tinnitus loudness of the CBT group was significantly higher than that of the Music group (*p* = 0.012), while no significant difference was found in Music-CBT vs. Music or Music-CBT vs. CBT. Details of individual subjects can be seen in Supplementary Tables S1–S5.

**Table 1:** Demographic and clinical characteristics among the three groups.

	Music-CBT (*n* = 22)	Music (*n* = 22)	CBT (*n* = 12)	*F*/*χ*^2^	*p*
Demographic characteristics
Age	35.82 (10.32)	37.41 (10.64)	42.83 (18.51)	1.241	0.297
Male gender	12 (55%)	12 (55%)	7 (58%)	0.055	0.973
Clinical characteristics
Tinnitus loudness (dB HL)	47.91 (16.74)	43.73 (11.60)	61 (20.72)	4.668	0.014*
Disease duration (months)	9.73 (7.86)	10.73 (6.37)	14.92 (14.24)	1.322	0.275
Hearing threshold (dB HL)	37.68 (18.17)	38.23 (12.25)	49.00 (24.41)	1.837	0.169
Tinnitus laterality (L/R/B)	10/8/4	11/6/6	2/6/4	3.149	0.207

*Indicates statistically significant.

### Behavioral Assessments

**Figure 1:**
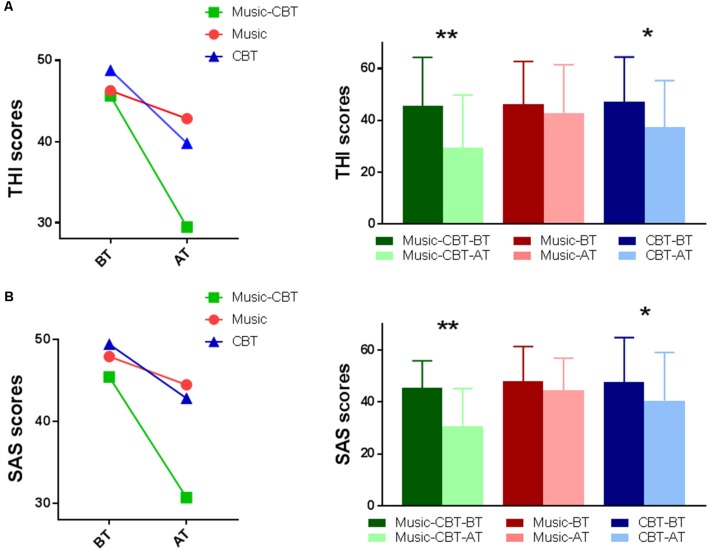
Split-Plot ANOVAs of the behavioral data. **(A)** THI results; **(B)** SAS results; Left: interaction effect of Group × Time Of Test. Right: simple effects of BT vs. AT for each group. *indicates a significance level of *p* < 0.025, and **Indicates *p* < 0.001. Abbreviations: CBT, cognitive behavioral therapy; BT, before-treatment; AT, after-treatment; THI, tinnitus handicap inventory; SAS, self-rating anxiety scale.

For the ANOVA of THI scores, Levene’s test showed no violation of homogeneity of variance (BT: *F*_(2,53)_ = 0.265, *p* = 0.768; AT: *F*_(2,53)_ = 0.410, *p* = 0.666). A significant main effect of TimeOfTest was found (*F*_(1,53)_ = 49.273, *P* < 0.001, $\eta_{\text{p}}^{2}$ = 0.482). A significant interaction effect of TimeOfTest × Group was also found (*F*_(2,53)_ = 9.174, *P* < 0.001, $\eta_{\text{p}}^{2}$ = 0.257), indicating a difference of performance among the groups between the two treatments (see the left panel of Figure 1A). To see where these differences were located, subsequent simple effects analysis was performed. The results showed significant differences in BT vs. AT for the Music-CBT group (mean difference = 16.182, SE = 2.131, *p* < 0.001 with a 95% confidence interval of 11.907–20.456) and CBT group (mean difference = 9.833, SE = 2.885, *p* = 0.001 with a 95% confidence interval of 4.046–15.621), whereas no difference was found for the Music group (mean difference = 3.273, SE = 2.885, *p* = 0.131 with a 95% confidence interval of −1.002 to 7.547; see the right panel of Figure 1A).

For the ANOVA of SAS scores, Levene’s test showed no violation of homogeneity of variance (BT: *F*_(2,53)_ = 1.951, *p* = 0.152; AT: *F*_(2,53)_ = 0.636, *p* = 0.533). A significant main effect of TimeOfTest was found (*F*_(1,53)_ = 45.186, *P* < 0.001, $\eta_{\text{p}}^{2}$ = 0.460). A significant interaction of TimeOfTest × Group was also found (*F*_(2,53)_ = 10.387, *P* < 0.001, $\eta_{\text{p}}^{2}$ = 0.282), indicating a difference of performance among the groups between the two treatments (see the left panel of Figure 1B). To see where these differences were located, subsequent simple effects analysis were performed. The results showed significant differences in BT vs. AT for the Music-CBT group (mean difference = 14.727, SE = 1.865, *p* < 0.001, with a 95% confidence interval of 10.987–18.468) and CBT group (mean difference = 7.000, SE = 2.525, *p* = 0.008, with a 95% confidence interval of 1.935–12.065), whereas no difference was found for the Music group (mean difference = 2.818, SE = 1.865, *p* = 0.137, with a 95% confidence interval of -0.92 to 6.559; see the right panel of Figure 1B).

### EEG Spectral Power Analysis

**Figure 2:**
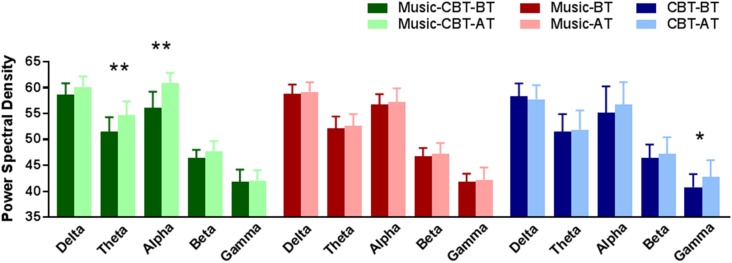
Simple effect analysis results of EEG band power changes in BT vs. AT for the three groups. *Indicates a significance level of *p* < 0.01, and **indicates *p* < 0.001. Abbreviations: CBT, cognitive behavioral therapy; BT, before-treatment; AT, after-treatment.

Levene’s test showed a violation of homogeneity of variance in the after-treatment theta values (*F*_(2,53)_ = 3.72, *p* = 0.03), before-treatment alpha values (*F*_(2,53)_ = 7.432, *p* < 0.01) and before-treatment gamma values (*F*_(2,53)_ = 4.91, *p* = 0.01), therefore Pillai’s criterion were used for the ANOVA analyses. Results of the ANOVAs for the five frequency bands are listed in Table 2. Significant main effects of TimeOfTest were found in the theta and alpha bands, and significant interaction effects of Group × TimeOfTest were found in theta, alpha and gamma bands. As shown in Table 2 and Figure 2, simple effect analyses found significantly increased theta and alpha values after the Music-CBT treatment only, and significantly increased gamma values after the CBT treatment only.

**Table 2:** Split-Plot ANOVAs of EEG band powers among the three groups.

	Main effect			Interaction effect			Simple effects (BT vs. AT)					
							Music-CBT		Music		CBT	
	*F*_(1,53)_	Sig.	$\eta_{\text{p}}^{2}$	*F*_(2,53)_	Sig.	$\eta_{\text{p}}^{2}$	MD (SE)	Sig.	MD (SE)	Sig.	MD (SE)	Sig.
δ	1.21	0.28	0.02	2.16	0.13	0.08	−1.46 (0.60)	0.02	−0.40 (0.60)	0.51	−0.58 (0.81)	0.48
θ	**7.606**	**0.01**	**0.13**	3.99	0.02	0.13	**−3.23 (0.77)**	**0.001**	−0.45 (0.77)	0.56	−0.46 (1.04)	0.66
α	**24.42**	**0.001**	**0.32**	**9.73**	**0.001**	**0.27**	**−4.84 (0.72)**	0.001	−0.45 (0.72)	0.53	−1.66 (0.97)	0.09
β	4.36	0.04	0.08	0.50	0.61	0.02	−1.34 (0.64)	0.04	−0.45 (0.64)	0.48	−0.80 (0.86)	0.36
γ	4.52	0.04	0.08	2.44	0.10	0.08	−0.30 (1.44)	0.84	−0.45 (1.44)	0.75	**−5.23 (1.95)**	**0.01**

*The bold data are results that are statistically significant (*P* < 0.01)*.

### Source Localization Analysis

**Figure 3:**
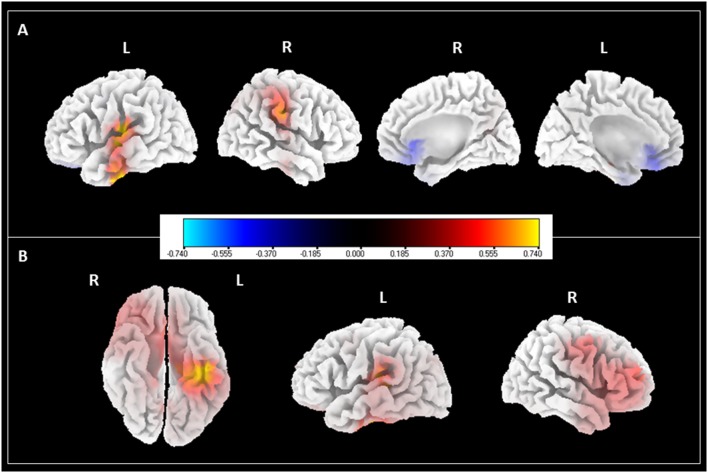
SLoreta analysis results for after-treatment > before-treatment comparison with Music-CBT. Results shown were thresholded at *p* < 0.05, uncorrected. Color bar indicates current density values. **(A)** Changes in alpha power. **(B)** Changes in theta power.

Given the significant difference found in the alpha and theta powers brought upon by the Music-CBT treatment, we performed source localization analysis on these data to further investigate the spatial patterns for these changes. SLoreta analysis results were shown in Figure 3. For the band, we found an increase from primarily the left superior temporal gyrus and right postcentral gyrus, as well as a decrease from the ventromedial prefrontal cortex (vMPFC)/pregenual anterior cingulate cortex (pgACC); for the theta band, increases were found in the right frontal regions as well as left hippocampus/parahippocampus.

## Discussion

The current study compared the outcomes from three different treatment approaches—Music-CBT, Music therapy, and CBT—on three groups of tinnitus patients. Behavioral assessments showed that for both the Music-CBT and CBT groups, symptom severities improved after 3 months of intervention, with the Music-CBT group showing seemingly more robust effects, while no significant improvement was seen for the Music group. Spectral power analysis on resting-state EEG data revealed an increase in alpha and theta band powers after the Music-CBT treatment, and an increase of gamma power after the CBT, while no significant difference was found for the Music treatment. Since reduced activities in alpha and theta were frequently found in tinnitus patients (Weisz et al., 2005, 2011; Roux et al., 2013; Hong et al., 2016), the increased powers after the Music-CBT treatment indicated an improvement on a neuronal level occurred due to the intervention. Combining the findings from both neural and behavioral data, this study showed that the Music-CBT treatment has an advantageous effect in improving tinnitus, with respect to CBT or Music treatment alone.

Further source localization analyses to alpha and theta changes in the Music-CBT group showed interesting information regarding the spatial pattern of these neural changes. First, two lateral views of the cortex in the left panel of Figure 3A showed the increase of alpha power generated in the auditory cortex. This supports several previous studies that indicated a correlation between auditory alpha decrease and tinnitus perception (Weisz et al., 2005, 2011; Schlee et al., 2009; Mazaheri et al., 2014). It is suggested that alpha waves might reflect the neuronal activities of cortical inhibition, where a low level of alpha activity might reflect a state of excitation (Hanslmayr et al., 2007, 2013; Klimesch et al., 2007; van Dijk et al., 2008; Weisz et al., 2011). In tinnitus patients, auditory alpha activity reduction (Weisz et al., 2005; Schlee et al., 2009, 2014) as well as hyperactivities in the auditory cortex (Arnold et al., 1996; Ochi and Eggermont, 1997; Chen et al., 2015b) are frequently found. This could be due to neuronal deafferentation from a peripheral damage contributing to a loss of balance between excitatory and inhibitory modulation, causing hyperactivities in the auditory cortex and thus the phantom perception (Eggermont and Roberts, 2004; Weisz et al., 2011). The increase of auditory alpha power after the Music-CBT treatment could indicate an improvement of this balance (i.e., increase of inhibition), thus an alleviation of the spontaneous firing of the auditory signals.

Second, two medial views of the cortex shown at the right panel in Figure 3A shows that, apart from increase of alpha power in the auditory cortex, there is also reduction of alpha power from vMPFC. Various researchers over the years have pointed out that auditory cortex might not be the sole concern underlying tinnitus (Jastreboff, 1990; Rauschecker et al., 2010; Leaver et al., 2011; Auerbach et al., 2014; De Ridder et al., 2014). Many authors suggested that a compromised limbic regulation could also be at play (Rauschecker et al., 2010; Leaver et al., 2011), given that acoustic features of the phantom sound (perceived loudness) and distress related to tinnitus could be two independent factors contributing to the sustaining of the disorder (Meyer et al., 2014, 2017). It is suggested that a limbic-auditory interaction could have constituted a “noise-cancelling” system, comprising mainly nodes of the thalamus, nucleus accumbens (NAc) and vMPFC, in which the vMPFC specializes in orienting attentional and emotional modulation into inhibiting the unwanted sensory signals through NAc and thalamus (Rauschecker et al., 2010; Leaver et al., 2011). Furthermore, activities in this region have been found to be related to processing the pleasantness of the stimuli (Sabatinelli et al., 2007; Junghofer et al., 2017), and animal studies have found that lesions to this region could induce depression-like behaviors in rat (Chang et al., 2014). VMPFC abnormalities have also been found in tinnitus patients with both structural (Mühlau et al., 2006; Leaver et al., 2011) and functional evidences (Seydell-Greenwald et al., 2012). For instance, one study found a positive correlation between alpha activities in vMPFC and distress symptoms in tinnitus patients (Vanneste et al., 2010). Combined with the finding that the generation of pleasantness is linked to the excitatory rather than inhibitory activities (Junghofer et al., 2017), our finding of the decreased alpha power in vMPFC could indicate a decreased inhibition and thus enhanced emotional regulation in the patients after the treatment (possibly by increased pleasantness signals and positive emotions). This is also substantiated by the improvement of behavioral assessments in especially the SAS ratings after the treatment. This enhanced emotional regulation could in turn strengthen the suppression of unwanted sensory signals, i.e., the phantom sounds.

Third, a bottom view of the cortex in Figure 3B showed the increase of theta power at the hippocampus/parahippocampus after the treatment. This could be due to the involvement of memory in tinnitus. Hippocampus/parahippocampus are found primarily in memory consolidation (van Strien et al., 2009), and reductions in their volume have been linked to depression (Colla et al., 2007; de Geus et al., 2007). Abnormality in these regions is also frequently found in tinnitus (Crippa et al., 2010; Salvi et al., 2011; Chen et al., 2015a). Evidences suggest a possible contribution of the hippocampus/parahippocampus to tinnitus, in retrieving auditory information from memory as a compensation for insufficient sensory input to resolve uncertainty (De Ridder et al., 2011), which could result in a consolidation of (negative) emotional memory associated with the tinnitus sound (De Ridder et al., 2006, 2011). Theta rhythms have been found to be characteristic for activities in the hippocampus/parahippocampus regions (Mitchell et al., 2008), and an increase of theta power in this region after treatment could indicate a normalization of auditory memory retrieval.

Fourth, two lateral views of the cortex in Figure 3B shows increased theta activities in primarily the lateral prefrontal cortex. This finding is consistent with previous findings, in that this region has been frequently found to be involved in executive control and working memory (Mars and Grol, 2007; Barbey et al., 2013), and phase-locked theta activities were found to be correlated with this region in top-down task performance (Phillips et al., 2014). Given that tinnitus patients have been found to have worse performance in multi-modal sensory tasks (visual and auditory), and showed a reduction in especially inhibitory executive control (Araneda et al., 2015), the increased theta activity in lateral prefrontal cortex found in our study could indicate an improvement of cognitive control for tinnitus patients due to the alleviation of symptoms after the Music-CBT treatment.

Several limitations to this article should be noted. First, the sample sizes in this study are generally small, which could have contributed to the difference found in tinnitus loudness among the groups. In particular, the unbalanced sample size of the CBT group (*n* = 12) with the other two groups (*n* = 22), could significantly limit the generalizability of the conclusions, especially regarding the increased gamma activity after treatment. Furthermore, for ecological considerations, procedures in all three treatments in our study were mostly self-administered by the participants themselves at home, which is common practice in current tinnitus treatments. The lack of laboratory control could introduce uncertainties into the findings. Combining the above two issues, findings in the current study regarding an advantageous effect in Music-CBT treatment should largely be considered preliminary, and further studies adopting larger sample sizes and strict laboratory control should be performed before any conclusive remarks could be made. Last, given the difficulty in solving the inverse problem in EEG, accuracy of source localization of EEG data is still unclear (Cuffin et al., 2001; Grech et al., 2008), and spatial resolution of EEG signals was not ideal. FMRI data is superior in such domain, and therefore is also recommended in future endeavors for a more targeted and precise investigation of the dynamic changes in the tinnitus neural network.

## Conclusion

Both the behavioral and EEG results in this study supported our hypothesis that the integrative Music-CBT treatment was advantageous in improving tinnitus with respect to music or CBT alone. The findings indicate that Music-CBT could be a robust approach in alleviating distress associated with tinnitus, which is reflected in increased theta and alpha powers in extensive brain regions encompassing both auditory cortex, and emotional/cognitive control regions.

## Data Availability Statement

The datasets used and analyzed during the current study are available from the corresponding author upon reasonable request.

## Ethics Statement

The studies involving human participants were reviewed and approved by Institution Review Board of the Sun Yat-sen Memorial Hospital of Sun Yat-sen University, China. The patients/participants provided their written informed consent to participate in this study.

## Author Contributions

TF was responsible for data collection, data processing and writing of the article. MW was responsible for data processing and writing of the article. HX, YZ, and HY were responsible for designing the experiment, writing and supervision of the research.

## Conflict of Interest

The authors declare that the research was conducted in the absence of any commercial or financial relationships that could be construed as a potential conflict of interest.
