# Supplementary material for: Efficacy of an Integrative Treatment for Tinnitus Combining Music and Cognitive-Behavioral Therapy—Assessed With Behavioral and EEG Data
Source: Front Integr Neurosci. 2020 Apr 7;14:12. doi: 10.3389/fnint.2020.00012 (PMC7155387; doi:10.3389/fnint.2020.00012)
Supplement: Supplementary file 1 [file Data_Sheet_1.docx]

Supplementary Material

# Supplementary Figure1. Locations for the HydroCel Geodesic Sensor Net electrodes.

#
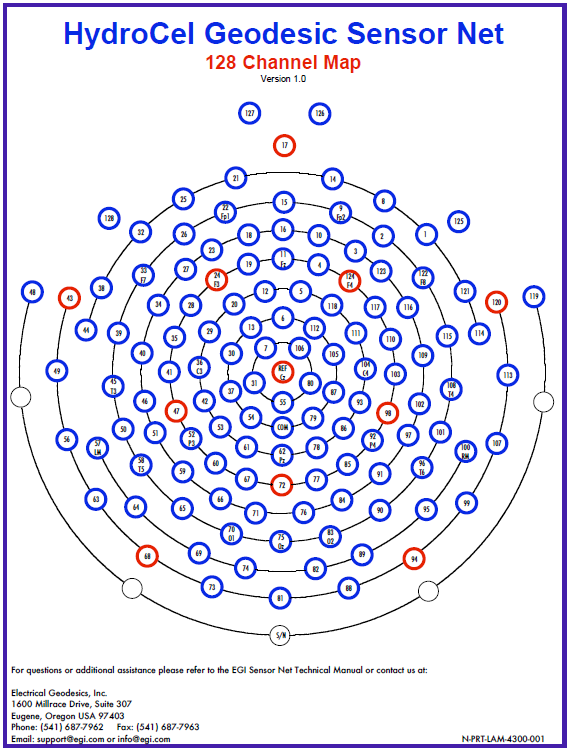


# Supplementary Table1. Demographic and clinical status of subjects in the Music-CBT group.

| **Num** | **Gender** | **Age** | **Tinnitus laterality** | **Duration (months)** | **Tinnitus pitch (Hz)** | **Tinnitus loudness (dBHL)** | **Hearing threshold (dBHL)** |
| --- | --- | --- | --- | --- | --- | --- | --- |
| ID1 | M | 39 | Left | 36 | 125 | 30 | 27 |
| ID2 | M | 41 | Right | 12 | 250 | 36 | 30 |
| ID3 | M | 35 | Right | 14 | 1000 | 46 | 20 |
| ID4 | M | 29 | Right | 12 | 8000 | 62 | 55 |
| ID5 | M | 32 | Right | 4 | 4000 | 54 | 45 |
| ID6 | M | 16 | Left | 3 | 2000 | 60 | 50 |
| ID7 | F | 43 | Left | 3 | 8000 | 28 | 35 |
| ID8 | M | 53 | Right | 3 | 125 | 43 | 47 |
| ID9 | F | 29 | Bilateral | 3 | 8000 | 66/64 | 73/70 |
| ID10 | M | 31 | Left | 4 | 1500 | 31 | 21 |
| ID11 | F | 34 | Right | 8 | 500 | 44 | 20 |
| ID12 | F | 43 | Bilateral | 12 | 6000 | 34/36 | 30/18 |
| ID13 | F | 59 | Bilateral | 24 | 125 | 63/56 | 49/64 |
| ID14 | F | 45 | Left | 6 | 3000 | 40 | 20 |
| ID15 | F | 33 | Left | 5 | 8000 | 52 | 63 |
| ID16 | F | 26 | Left | 6 | 4000 | 49 | 32 |
| ID17 | F | 29 | Left | 15 | 8000 | 58 | 30 |
| ID18 | M | 34 | Left | 12 | 6000 | 86 | 70 |
| ID19 | F | 31 | Right | 8 | 3000 | 16 | 8 |
| ID20 | M | 25 | Right | 6 | 250 | 61 | 46 |
| ID21 | M | 53 | Bilateral | 6 | 4000 | 22/34 | 12/19 |
| ID22 | M | 28 | Bilateral | 12 | 8000 | 70/70 | 42/40 |

**Supplementary Table2.** Demographic and clinical status of subjects in the Music group.

| **Num** | **Gender** | **Age** | **Tinnitus laterality** | **Duration (months)** | **Tinnitus pitch (Hz)** | **Tinnitus loudness (dBHL)** | **Hearing threshold (dBHL)** |
| --- | --- | --- | --- | --- | --- | --- | --- |
| ID1 | M | 35 | Left | 20 | 250 | 30 | 20 |
| ID2 | F | 45 | Bilateral | 5 | 8000 | 40/36 | 37/26 |
| ID3 | M | 35 | Left | 8 | 4000 | 37 | 47 |
| ID4 | M | 28 | Right | 11 | 6000 | 54 | 45 |
| ID5 | F | 56 | Left | 6 | 125 | 49 | 40 |
| ID6 | M | 31 | Left | 5 | 8000 | 66 | 68 |
| ID7 | F | 30 | Left | 7 | 6000 | 30 | 23 |
| ID8 | F | 44 | Left | 6 | 125 | 32 | 33 |
| ID9 | M | 30 | Left | 6 | 250 | 55 | 47 |
| ID10 | M | 43 | Right | 18 | 3000 | 45 | 28 |
| ID11 | F | 24 | Right | 24 | 500 | 44 | 30 |
| ID12 | M | 36 | Bilateral | 12 | 4000 | 60/64 | 55/56 |
| ID13 | M | 29 | Left | 9 | 125 | 52 | 44 |
| ID14 | F | 33 | Right | 12 | 6000 | 40 | 40 |
| ID15 | M | 29 | Bilateral | 6 | 8000 | 52/60 | 36/52 |
| ID16 | F | 38 | Left | 24 | 4000 | 45 | 37 |
| ID17 | M | 66 | Left | 20 | 250 | 30 | 48 |
| ID18 | F | 38 | Bilateral | 5 | 8000 | 40/36 | 46 |
| ID19 | M | 38 | Right | 12 | 8000 | 45 | 30 |
| ID20 | F | 40 | Left | 5 | 4000 | 36 | 28 |
| ID21 | F | 22 | Right | 9 | 4000 | 56 | 40 |
| ID22 | M | 53 | Bilateral | 6 | 250 | 22/22 | 15/15 |

**Supplementary Table3.** Demographic and clinical status of subjects in the CBT group.

| **Num** | **Gender** | **Age** | **Tinnitus laterality** | **Duration (months)** | **Tinnitus pitch (Hz)** | **Tinnitus loudness (dBHL)** | **Hearing threshold (dBHL)** |
| --- | --- | --- | --- | --- | --- | --- | --- |
| ID1 | F | 22 | Right | 3 | 1000 | 56 | 50 |
| ID2 | M | 50 | Right | 7 | 1000 | 75 | 73 |
| ID3 | M | 67 | Right | 36 | 1000 | 70 | 65 |
| ID4 | F | 66 | Bilateral | 12 | 8000 | 68/98 | 66/90 |
| ID5 | F | 26 | Left | 24 | 6000 | 61 | 24 |
| ID6 | F | 54 | Right | 5 | 1000 | 80 | 74 |
| ID7 | M | 60 | Right | 6 | 1000 | 73 | 75 |
| ID8 | M | 38 | Bilateral | 6 | 8000 | 39/29 | 24/16 |
| ID9 | M | 28 | Left | 18 | 3000 | 63 | 50 |
| ID10 | F | 24 | Bilateral | 6 | 8000 | 10/10 | 6//8 |
| ID11 | M | 60 | Bilateral | 48 | 3000 | 57/57 | 35/41 |
| ID12 | M | 19 | Right | 8 | 8000 | 70 | 34 |

**Supplementary Table4. Tinnitus handicap inventory scores of the three patient groups.**

|  | **Music-CBT** | | **Music** | | **CBT** | |
| --- | --- | --- | --- | --- | --- | --- |
|  | **BT** | **AT** | **BT** | **AT** |  |  |
| **1** | 46 | 48 | 78 | 94 | 30 | 20 |
| **2** | 72 | 60 | 28 | 26 | 40 | 38 |
| **3** | 86 | 68 | 70 | 70 | 42 | 24 |
| **4** | 28 | 20 | 10 | 16 | 68 | 60 |
| **5** | 38 | 20 | 64 | 58 | 80 | 74 |
| **6** | 28 | 0 | 28 | 24 | 40 | 32 |
| **7** | 74 | 42 | 22 | 22 | 72 | 60 |
| **8** | 26 | 10 | 62 | 54 | 48 | 32 |
| **9** | 50 | 12 | 52 | 58 | 44 | 38 |
| **10** | 60 | 50 | 40 | 20 | 24 | 20 |
| **11** | 56 | 14 | 44 | 44 | 44 | 20 |
| **12** | 30 | 12 | 42 | 30 | 34 | 30 |
| **13** | 26 | 20 | 40 | 38 |  |  |
| **14** | 44 | 40 | 42 | 36 |  |  |
| **15** | 36 | 26 | 44 | 36 |  |  |
| **16** | 56 | 40 | 42 | 36 |  |  |
| **17** | 68 | 66 | 32 | 28 |  |  |
| **18** | 24 | 24 | 48 | 48 |  |  |
| **19** | 62 | 22 | 62 | 56 |  |  |
| **20** | 28 | 12 | 50 | 44 |  |  |
| **21** | 26 | 0 | 60 | 60 |  |  |
| **22** | 40 | 42 | 58 | 44 |  |  |
| **Mean** | 45.64 | 29.45 | 46.27 | 42.82 | 47.17 | 37.33 |
| **SD** | 18.677 | 20.34 | 18.68 | 18.65 | 17.28 | 18.00 |

**Abbreviations:** CBT – cognitive-behavioral therapy; BT – before treatment; AT – after treatment; SD – standard deviation.

**Supplementary Table4. Subjective Anxiety Scale scores of the two patient groups.**

|  | **Music-CBT** | | **Music** | | **CBT** | |
| --- | --- | --- | --- | --- | --- | --- |
|  | **BT** | **AT** | **BT** | **AT** | **BT** | **AT** |
| **1** | 40 | 40 | 65 | 62 | 26 | 18 |
| **2** | 58 | 46 | 52 | 57 | 50 | 44 |
| **3** | 51 | 42 | 78 | 70 | 36 | 26 |
| **4** | 36 | 29 | 43 | 47 | 62 | 56 |
| **5** | 41 | 26 | 62 | 50 | 78 | 74 |
| **6** | 45 | 28 | 32 | 36 | 52 | 42 |
| **7** | 53 | 36 | 30 | 32 | 78 | 72 |
| **8** | 24 | 12 | 63 | 52 | 44 | 34 |
| **9** | 42 | 16 | 48 | 40 | 38 | 36 |
| **10** | 50 | 36 | 33 | 22 | 30 | 26 |
| **11** | 44 | 14 | 52 | 54 | 36 | 20 |
| **12** | 32 | 22 | 52 | 40 | 40 | 38 |
| **13** | 36 | 22 | 48 | 48 |  |  |
| **14** | 52 | 36 | 34 | 30 |  |  |
| **15** | 46 | 40 | 34 | 34 |  |  |
| **16** | 54 | 36 | 28 | 24 |  |  |
| **17** | 64 | 62 | 36 | 36 |  |  |
| **18** | 32 | 30 | 56 | 54 |  |  |
| **19** | 50 | 20 | 48 | 44 |  |  |
| **20** | 33 | 26 | 44 | 38 |  |  |
| **21** | 56 | 0 | 52 | 54 |  |  |
| **22** | 60 | 56 | 64 | 54 |  |  |
| **Mean** | 45.41 | 30.68 | 47.91 | 44.45 | 47.5 | 40.5 |
| **SD** | 10.44 | 14.43 | 13.41 | 12.36 | 17.29 | 18.55 |

**Abbreviations:** CBT – cognitive-behavioral therapy; BT – before treatment; AT – after treatment; SD – standard deviation.
